# Screening for Cardiovascular Disease in Pregnancy: Is There a Need?

**DOI:** 10.3390/jcdd9030089

**Published:** 2022-03-17

**Authors:** Melissa E. Chambers, Madushka Y. De Zoysa, Afshan B. Hameed

**Affiliations:** 1 Department of Obstetrics and Gynecology, University of California Irvine, Orange, CA 92868, USA; 2 Department of Maternal Fetal Medicine, University of California Irvine, Orange, CA 92868, USA; mdezoysa@hs.uci.edu (M.Y.D.Z.); ahameed@hs.uci.edu (A.B.H.)

**Keywords:** cardiovascular disease, pregnancy, maternal mortality, cardiomyopathy

## Abstract

Maternal mortality in the United States has been on the rise. Every year, about 700 women die from pregnancy-related complications. Cardiovascular disease (CVD) accounts for a large majority of pregnancy-related deaths driven by the lack of recognition and delays in diagnosis due to the overlap of normal pregnancy symptoms with those of CVD. Risk factors for CVD including race, advanced maternal age, hypertension, diabetes, obesity, socioeconomic status, and geographic region play an important role in CVD-related deaths. Several risk assessment models are available to stratify women with a known diagnosis of CVD. However, most women who die from CVD during pregnancy or the postpartum period do not have a prior diagnosis of CVD, and cardiomyopathy is an important contributor. The California Maternal Quality Care Collaborative (CMQCC) developed an algorithm to screen all pregnant and postpartum women to allow stratification into low or high risk for CVD. The algorithm has been validated in diverse patient populations. We propose universal CVD screening for all women in the antepartum and postpartum period to identify women at risk and to provide education and awareness for both patients and healthcare providers. This screening tool would work to reduce the increasing rates of severe maternal mortality and morbidity while having a significant impact on healthcare costs in the United States.

## 1. Introduction

**Figure 1:**
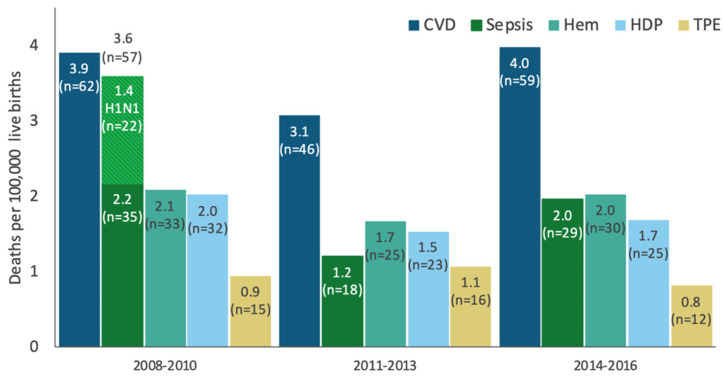
Pregnancy-related mortality ratio by cause, California 2008–2016.

In the United States, 700 women die annually from pregnancy-related complications [1]. Between 2014 and 2017, cardiomyopathy and other cardiovascular conditions accounted for 27% of all pregnancy-related deaths in the United States [2]. In California, cardiovascular disease (CVD) was responsible for 28% of all deaths among pregnant women between 2008 and 2016, with over half of these deaths due to cardiomyopathy [3]. Data collected through the California Pregnancy Mortality Surveillance System (CA-PMSS) demonstrates CVD has remained the leading cause of pregnancy-related mortality throughout this period (Figure 1). Review of the timing of CVD deaths deserves attention. Only 19% of CVD-related deaths occurred in the antepartum period (19%). The vast majority (>80%) of maternal deaths are encountered in the late postpartum period. The largest number of maternal deaths are seen beyond the first week postpartum, i.e., 6 days postpartum (24%), between 7 and 42 days postpartum (24%), and within 43–365 days postpartum (33%) [3]. Maternal deaths after 42 days postpartum are primarily driven by cardiomyopathy. Maternal mortality represents the tip of the iceberg. The CDC reports that maternal morbidity and mortality has increased 200% from 49.5 in 1993 to 144.0 in 2014 [4]. The etiology is multifactorial, which has led to extended length of hospitalization, increased costs, and higher utilization of healthcare services [5].

## 2. Cardiovascular Changes in Pregnancy

**Figure 2:**
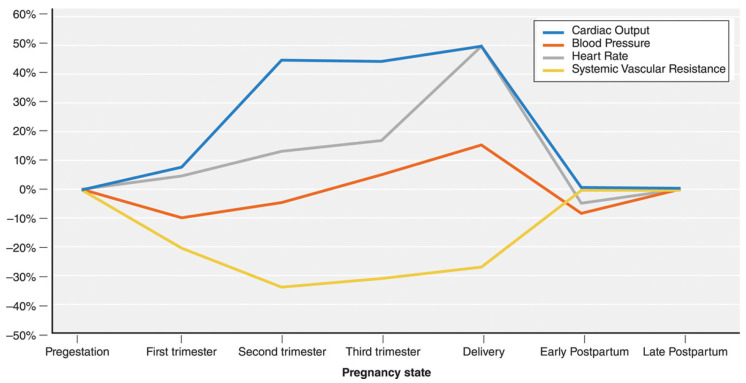
Physiologic changes during pregnancy. Mehta et al. Cardiovascular considerations in caring for pregnant patients, Circulation 2020 [7].

Pregnancy involves dramatic changes in cardiovascular physiology to promote increased perfusion to the uterus to meet the growing needs of the fetal–placental unit. Pregnancy-related increase in catecholamines, estrogen, and progesterone cause activation of the renin–angiotensin system that augment the cardiac output and plasma volume [6]. Other cardiovascular changes include increases in heart rate, decrease in diastolic blood pressure, increased RBC volume, and a decrease in systemic vascular resistance (Figure 2) [7]. Additionally, these physiologic changes lead to known cardiac structural and functional changes, i.e., increase in left ventricular end diastolic volume as well as ventricular mass demonstrated by echocardiography [6]. This may also result in sign and symptoms in a normal pregnancy that are like that of CVD and, therefore, diagnosis of CVD may be challenging. Not surprisingly, pregnancy is often considered a cardiovascular stress test that may worsen the pre-existing CVD or unmask a previously undiagnosed but well-compensated cardiac condition. 

### Pregnancy vs. Cardiovascular Disease 

Normal physiological changes in pregnancy lead to signs and symptoms that may be indistinguishable from those of CVD. Common symptoms of pregnancy include palpitations, shortness of breath, fatigue, chest pain, and dizziness. A systematic approach is required to differentiate normal pregnancy symptoms (Table 1). Based on patient-reported symptoms, vital signs, and physical exam findings (Table 1), providers can give reassurance or triage patients for further CVD evaluation with laboratory tests (BNP, troponin) and/or other diagnostic testing (EKG, chest radiography, stress test). It is not surprising that most women who died of CVD during pregnancy and/or the postpartum period were not suspected of having a cardiac diagnosis and symptoms were attributed to an alternate diagnosis. Roughly 84% of pregnant patients who died from CVD presented with symptoms concerning for cardiopulmonary disease. However, only 61.1% of these patients were referred to Cardiology, and, of those, only 7% were referred antenatally. The overlap of signs and symptoms of normal pregnancy with those of CVD further complicates timely diagnosis. About 60.9% of CVD-related maternal deaths were found to be due to delayed response from healthcare providers [8].

A key challenge for the healthcare providers who evaluate pregnant and postpartum women is to differentiate the sign and symptoms of normal pregnancy from those of CVD, particularly beyond the conventional postpartum period up to a year after delivery. 

## 3. Pregnancy-Related Cardiovascular Disease

Pregnancy-related CVD can be classified into two groups: (i) women with known pre-existing CVD and (ii) women without known pre-existing CVD. Women with known CVD include congenital heart disease, cardiomyopathy, valvular heart disease, arrhythmia, pulmonary hypertension, coronary artery disease, etc. [8]. This group of women is typically managed optimally by maternal fetal medicine specialists and cardiologists. Their mortality risk profile during pregnancy can be estimated using existing cardiovascular risk assessment tools that have been validated. Women without known pre-existing CVD can either develop a new diagnosis of CVD in pregnancy, i.e., peripartum cardiomyopathy, or CVD can be unmasked by pregnancy or a new diagnosis of CVD. This specific population of women is particularly at higher risk during pregnancy as conditions such as hypertension, diabetes, obesity and advanced age often coexist. The number of patients that fall into this category is large. In a review of CVD deaths in California between 2006 and 2006, only 3.1% of deaths due to CVD had a pre-existing CVD diagnosis, while most diagnoses (48.4%) were made postmortem [8]. Additionally, 64.1% of patients who died from pregnancy-related CVD had an underlying medical condition [8]. This suggests that a substantial proportion of these patients experienced either an exacerbation of an underlying condition leading to a CVD-related death or developed new peripartum cardiomyopathy, given that two-thirds of all CVD-related deaths were from cardiomyopathy [8].

It is not yet standard of care for obstetric providers to incorporate a robust screening and management strategy into the prenatal care of pregnant patients without existing cardiovascular disease. Additionally, as mentioned previously, differentiating normal pregnancy symptoms with CVD symptoms remains a challenge for providers. Due to these existing gaps in healthcare, provider misdiagnosis leads to delays in treatment and accounts for 26% of maternal CVD-related deaths, 37% of cardiomyopathy deaths, and 62% of preeclampsia/eclampsia-related deaths [9]. However, since we know that pregnancy increases a woman’s risk of developing CVD, a universal screening plan for all pregnant and postpartum women ought to be implemented and clinicians (obstetricians, gynecologists, and cardiologists) need to improve their knowledge of cardiovascular changes and complications in pregnancy in order to prevent adverse maternal outcomes.

## 4. Risk Factors Including Racial, Geographic, and Socioeconomic Disparities

Several risk factors have been identified as affecting CVD-related maternal mortality. These include race, advanced maternal age, hypertension, diabetes, obesity, geographic region, income, education level, and type of insurance [6,10,11]. Black women have been shown to be at a significant risk of not only CVD-related maternal mortality but also all-cause maternal mortality [3]. In a report from the nine maternal mortality review committees, 48.2% of pregnancy-related deaths were in Black women compared to 30.2% in Hispanic women and 28.4% in White women [12]. In California between 2008 and 2010, the pregnancy-related mortality ratio for Black women was more than three times higher than that of other racial groups [3]. By 2014–2016, this disparity was further widened as the mortality ratio for Black women was four to six times higher than that of other racial groups [3]. Specifically for CVD, Black women are 3.4 times more likely to die from CVD in pregnancy than White women [6]. Black women are more likely to have a delay in diagnosis of peripartum cardiomyopathy resulting in more severe disease (decrease ejection fraction) and poorer prognosis compared to non-Black women [13]. This is suggestive of not just a barrier for Black patients’ ability to access care, but also inherent flaws in the healthcare system fostering bias and racism that can result in missed diagnoses [6]. These findings mirror those demonstrated in a review of cardiovascular deaths in California between 2002 and 2006 [8]. Patients who died from CVD were more likely to be Black compared to those that died from non-CVD causes. Obesity, diabetes, and hypertension were also noted to be the most prevalent conditions in women who died from CVD [8]. 

In the United States between 2016 and 2019, a serial cross-section analysis of maternal birth records demonstrated a large geographic and socioeconomic disparity preventing access to equitable healthcare for pregnant women [11]. Favorable cardiometabolic health (normal BMI, no diabetes or hypertension) in pregnant women declined significantly, with the largest decline being in the Midwest and the South compared to the West and North East regions [11]. Additionally, the higher the prevalence of high school educated women in a region, the less favorable the cardiometabolic health outcomes [11]. This trend can also be attributed to insurance type as well: the higher the number of women enrolled in Medicaid, the less favorable the cardiometabolic health outcomes [11]. 

Among the multitude of risk factors seen during pregnancy, of which obesity and hypertension play a major role, and the inequities of health care access faced by racial minorities and persons of lower socioeconomic/education status, there is a stark under-diagnosis of CVD which is likely contributing to the high rates of CVD-related maternal mortality in this country. Consequently, the American College of Obstetricians and Gynecologists (ACOG) endorses universal CVD screening for all women in the antepartum and postpartum period, specifically using the CVD in Pregnancy and Postpartum Toolkit Algorithm [6,14].

## 5. Universal Cardiovascular Risk Assessment in Pregnancy and Postpartum Period

Pregnant and postpartum patients with CVD fall into two groups: (i) women with known pre-existing CVD and (ii) women without known pre-existing CVD that is either unmasked by pregnancy or a new diagnosis of CVD, i.e., peripartum cardiomyopathy. The existing cardiovascular risk assessment models that stratify women with a known diagnosis of CVD are being routinely used for preconception counseling and during pregnancy. These provide guidance to the healthcare provider to describe the level of risk involved, anticipated complications, level of maternal care requirements and frequency of planned visits with the cardiologist. Commonly used models include Modified WHO classification, CARPREG II, and Zahara predictors that have been validated in various studies [15,16,17]. While these tools are useful in directing management for patients with specific and pre-existing cardiac lesions, they are not applicable to the general obstetric population. Mortality reviews indicated that most women who die from CVD during pregnancy or the postpartum period do not have a prior diagnosis of CVD. Cardiac diagnosis is not considered in the differential diagnosis in a woman presenting with the normal symptoms of pregnancy such as shortness of breath or fatigue that often leads to delays in recognition and treatment. Early recognition of CVD will help triage women at risk to initiate appropriate timely treatment to prevent maternal morbidity and/or mortality. Most important is a new diagnosis of CVD during pregnancy, i.e., peripartum cardiomyopathy, that presents in the later part of pregnancy or in the postpartum period. Therefore, there is a need for a universal screening tool to assess all pregnant and postpartum patients for their risk of CVD. The proposed CVD screening tool should be easy to administer and applicable to all clinical settings. 

**Figure 3:**
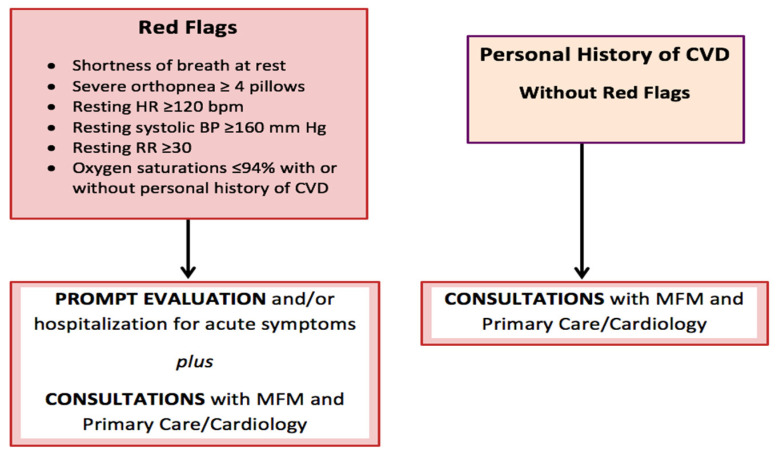
California Maternal Quality Care Collaborative (CMQCC) algorithm to identify red flags in pregnant and postpartum patients that would prompt further cardiovascular disease evaluation.

**Figure 4:**
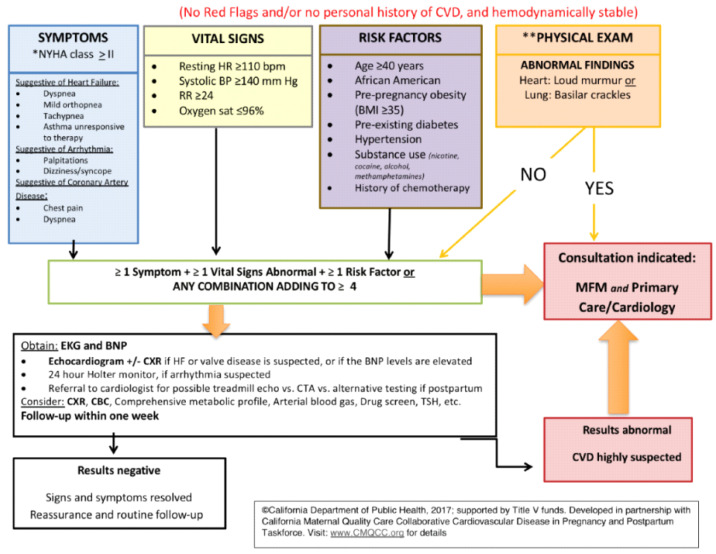
California Maternal Quality Care Collaborative (CMQCC) algorithm to identify pregnant and postpartum patients without red flags or personal history of CVD who are at high or low risk of CVD. * New York Hospital Association Functional Classification. ** Physical exam limited to heart (diastolic or systolic murmur) and lung (crackles, jugular venous distension, cyanosis, clubbing) exam.

The California Maternal Mortality Review Committee put together a tool kit that contains two CVD screening algorithms published by the California Maternal Quality Care Collaborative (CMQCC). The first algorithm (Figure 3) calls for “red flags” identified as the patient’s reported severe symptoms or severe vital sign abnormalities, and for patients with known CVD, i.e., prior history of CVD. Presence of any of these three elements guides the clinician to perform further cardiac testing and prompt evaluation. The second algorithm (Figure 4), however, applies to patients without red flags or personal history of CVD. It uses demographics, self-reported symptoms, vital sign abnormalities and abnormal physical examination findings to stratify pregnant/postpartum women into high vs. low risk of CVD. If the patient screens positive for CVD, it warrants further workup with an EKG, BNP, and/or other diagnostic tests. Additionally, consultation with maternal fetal medicine and/or cardiology is recommended [14]. If results are negative, the patient simply receives reassurance and routine obstetric follow up is continued. This second screening algorithm was validated in women who had died of CVD. It was demonstrated that 88% of cases would have been identified as high risk of CVD in asymptomatic women and 93% of symptomatic women that would have prompted further CVD testing [8]. 

### CVD Toolkit Application and Future Implications

Blumenthal et.al. reported on prospective screening of pregnant and postpartum women at two large academic centers in California and New York using the CMQCC CVD algorithm. The screening algorithm identified 8% of women at both sites as screen-positive, i.e., at increased risk for CVD. At both sites, combinations of moderate risk factors were the driving force behind positive screens rather than “red flag” signs alone. Between the two sites, New York had a higher screen-positive rate (19%) compared to California (5%). This may in part be due to the higher proportion of Black women enrolled in the study in New York (35%) compared to that in California (2.7%). Subsequent cardiovascular testing confirmed CVD in 34% of those patients who screened positive for CVD and completed follow up. Of note, a higher proportion of screen-positive patients completed all follow up studies in California compared to New York (70% vs. 27%, respectively). One proposed explanation for this finding was that the screening and testing were performed by the patients’ primary providers in California versus a separate research team in New York [18]. This suggests the physician–patient relationship may play a role regarding perceived importance of CVD risk assessment in patients, which is supported by the mortality review by Hameed et al. where both lack of patient knowledge and lack of provider continuity were identified as risk factors contributing to pregnancy-related cardiovascular deaths [8].

The finding that cumulative moderate risk factors rather than “red flags” led to more positive screens reinforces the assertion by Hameed et al. that most of the cardiovascular-related maternal mortality can be attributed to a delayed response and misdiagnosis on the part of the provider, particularly when almost all the moderate risk symptoms in the CVD algorithm are also common symptoms of pregnancy [8]. It is important to note that based on the study by Blumenthal et al., communication between the provider and patient is suggested to aid in achieving a higher chance of workup completion and subsequent identification of true-positive CVD patients. These lessons will be applied as the CVD algorithm is used for universal screening of all antenatal patients at our institution.

Integration of the CVD screening tool in the electronic medical record system is essential for rapid CVD screening in pregnant and postpartum patients. Studies looking to minimize the time spent by the healthcare provider to administer the CVD screen and linking the positive screen to the order sets prompting diagnostic testing and referrals are underway to address this important area of women’s heart health. Additional studies are ongoing to optimize the screening algorithm for use on a larger scale.

As previously stated, one of the barriers to CVD-related care is lack of knowledge at the healthcare provider and the patient level. Therefore, a key step in preventing maternal death can be reduced by addressing provider awareness and education surrounding CVD in pregnancy. Additionally, this universal screening tool would improve access to healthcare for pregnant women especially at a lower socioeconomic status and reduce the percentage of misdiagnoses and delay in treatment of CVD. In turn, early diagnosis and treatment of CVD has the potential to reduce healthcare costs in addition to significantly affect maternal mortality and morbidity in the United States [10]. One study evaluating the cost-effectiveness of cardiovascular screening performed by Smith et al. out of Colorado demonstrated a screening tool in combination with community health workers reaching out to individuals to implement interventions resulted in a 0.8% reduction in Framingham risk score in the general population and a 2.0% reduction in an at-risk population. In their cost analyses, they demonstrated not just an increase in quality-adjusted life-years (QALYs) but, more importantly, a significant savings in healthcare costs in both the general population as well as an at-risk population [19]. Given the large proportion of pregnancy-related mortality attributed to cardiovascular disease, these data suggest that an implementation of universal screening may be beneficial to overall healthcare costs in addition to improvement of overall maternal outcomes; however, a secondary analysis of healthcare-related costs would be indicated after implementation of our algorithm.

## 6. Conclusions

Most of the deaths from CVD in pregnancy are attributed to acquired, as opposed to congenital, heart disease [6]. Maternal mortality reviews estimate that CVD-related deaths could be prevented through early diagnosis and treatment [8]. Moreover, half of the serious cardiac complications are preventable in women with cardiac disease [20,21]. Studies demonstrate that conventional CVD risk factors such as age, hypertension, diabetes, obesity, etc., play a significant role in CVD-related mortality [22]. Identification of these risk factors in conjunction with universal CVD screening to assist in early diagnosis is essential in efforts to reduce maternal mortality. We propose universal CVD screening for all women in the antepartum and postpartum period to identify women at risk and to provide education and awareness for both patients and healthcare providers.

## Figures and Tables

**Table 1:** How to differentiate common signs and symptoms of normal pregnancy versus those that are abnormal and indicative of underlying cardiac disease.

	ROUTINE CARE	CAUTION	STOP
	Reassurance	Nonemergent Evaluation	Prompt Evaluation
History of CVD	None	None	Yes
Self-Reported Symptoms	None or mild	Yes	Yes
Shortness of breath	No interference with activities of daily living; with heavy exertion only	Yes; with moderate exertion, new onset asthma, persistent cough or moderate/severe OSA	Yes, at rest; paroxysmal nocturnal dyspnea or orthopnea, bilateral chest infiltrates or refractory pneumonia
Chest pain	Reflux-related that resolves with treatment	Atypical	At rest or with minimal exertion
Palpitations	Few seconds, self-limited	Brief, self-limited episode, no light-headedness or syncope	Associated with near syncope
Syncope	Dizziness only with prolonged standing or dehydration	Vasovagal	Exertion or unprovoked
Fatigue	Mild	Mild or moderate	Extreme
Vital Signs	Normal		
HR (bpm)	<90	90–119	≥120
Systolic BP (mm Hg)	120–139	140–159	≥160 (or symptomatic low blood pressure)
RR (per minute)	12–15	16–25	≥25
Oxygen Saturation	>97%	95–97%	<95% (unless chronic)
Physical Exam	Normal		
JVP	Not visible	Not visible	Visible >2 cm above clavicle
Heart	S3 barely audible soft systolic murmur	S3 systolic murmur	Loud systolic murmur, diastolic murmur S4
Lungs	Clear	Clear	Wheezing, crackles, effusion
Edema	Mild	Moderate	Marked

Practice Bulletin 2019, Pregnancy and Heart Disease, ACOG.

## Data Availability

Not applicable.
